# Bone Strain Index predicts fragility fracture in osteoporotic women: an artificial intelligence-based study

**DOI:** 10.1186/s41747-021-00242-0

**Published:** 2021-10-19

**Authors:** Fabio Massimo Ulivieri, Luca Rinaudo, Carmelo Messina, Luca Petruccio Piodi, Davide Capra, Barbara Lupi, Camilla Meneguzzo, Luca Maria Sconfienza, Francesco Sardanelli, Andrea Giustina, Enzo Grossi

**Affiliations:** 1 Fondazione IRCCS Ca’ Granda Ospedale Maggiore Policlinico, Via Francesco Sforza, 35, 20122 Milan, Italy; 2 Current address: Università Vita-Salute San Raffaele, Via Olgettina, 58 20132 Milan, Italy; 3 BSE TECHNOLOGIC S.r.l., Lungo Dora Voghera, 34/36A, 10153 Turin, Italy; 4 IRCCS Istituto Ortopedico Galeazzi, Via Riccardo Galeazzi, 4, 20161 Milan, Italy; 5 Former: Gastroenterology and Digestive Endoscopy Unit, Fondazione IRCCS Ca’ Granda Ospedale Maggiore Policlinico, Via Francesco Sforza, 35, 20122 Milan, Italy; 6 Dipartimento di Scienze Biomediche per la Salute, Università degli Studi di Milano, Via Pascal, 36, 20133 Milan, Italy; 7 Scuola di Specializzazione in Medicina Fisica e Riabilitativa, Università degli Studi di Milano, Via Festa del Perdono, 7, 20122 Milan, Italy; 8 IRCCS Policlinico San Donato, Via Rodolfo Morandi, 30, 20097 San Donato Milanese, Milan Italy; 9 Institute of Endocrine and Metabolic Sciences (IEMS) San Raffaele Vita-Salute University, IRCCS San Raffaele Hospital, Via Olgettina Milano, 20, 20132 Milan, MI Italy; 10 Villa Santa Maria Foundation, Via IV Novembre, 15, 22038 Tavernerio, Como Italy

**Keywords:** Neural network models, Dual-energy x-ray absorptiometry, Finite element analysis, Artificial intelligence, Osteoporosis

## Abstract

**Background:**

We applied an artificial intelligence-based model to predict fragility fractures in postmenopausal women, using different dual-energy x-ray absorptiometry (DXA) parameters.

**Methods:**

One hundred seventy-four postmenopausal women without vertebral fractures (VFs) at baseline (mean age 66.3 ± 9.8) were retrospectively evaluated. Data has been collected from September 2010 to August 2018. All subjects performed a spine x-ray to assess VFs, together with lumbar and femoral DXA for bone mineral density (BMD) and the bone strain index (BSI) evaluation. Follow-up exams were performed after 3.34 ± 1.91 years. Considering the occurrence of new VFs at follow-up, two groups were created: fractured *versus* not-fractured. We applied an artificial neural network (ANN) analysis with a predictive tool (TWIST system) to select relevant input data from a list of 13 variables including BMD and BSI. A semantic connectivity map was built to analyse the connections among variables within the groups. For group comparisons, an independent-samples *t*-test was used; variables were expressed as mean ± standard deviation.

**Results:**

For each patient, we evaluated a total of *n* = 6 exams. At follow-up, *n* = 69 (39.6%) women developed a VF. ANNs reached a predictive accuracy of 79.56% within the training testing procedure, with a sensitivity of 80.93% and a specificity of 78.18%. The semantic connectivity map showed that a low BSI at the total femur is connected to the absence of VFs.

**Conclusion:**

We found a high performance of ANN analysis in predicting the occurrence of VFs. Femoral BSI appears as a useful DXA index to identify patients at lower risk for lumbar VFs.

## Key points

- Bone strain index (BSI) is a new finite element analysis Dual-energy x-ray absorptiometry-based parameter.
- Artificial neural network analysis showed high performance in predicting osteoporotic vertebral fractures.
- Femoral BSI appears useful in identifying patients at a lower risk for lumbar fractures.

## Background

Osteoporosis is a metabolic bone disease characterised by a reduction in bone mass and deterioration in the texture and architecture of the bone, leading to fragility fractures [1].

In clinical practice, the diagnosis of osteoporosis is based on the measurement of bone mineral density (BMD) by dual-energy x-ray absorptiometry (DXA) [2]. Areal BMD is responsible for about two-thirds of bone strength, and fracture risk increases proportionally with the reduction of BMD [3]. Nevertheless, BMD measurements alone are not fully capable of detecting fracture risk, as an overlap of BMD values exists between patients with or without fractures [4]. Therefore, there is a need to evaluate other factors that can predict fracture risk in addition to BMD, such as bone microarchitecture and textural structure [5]. Such evaluation can be done invasively with bone biopsy or with imaging techniques such as high-resolution peripheral quantitative computed tomography or magnetic resonance imaging [6, 7]. These techniques are, however, expensive or expose a high ionising radiation dose to the patient, and therefore not accessible for screening. For these reasons, other DXA indexes have been developed for bone micro-architecture analysis. An example is the Trabecular Bone Score (TBS), a lumbar spine DXA-derived tool that correlates with histomorphometric bone parameters [8]. Previous studies showed that the TBS can predict the fracture risk partially independent from BMD [9, 10].

Recently, a new DXA-based structural parameter has been introduced with the name of Bone Strain Index (BSI). This tool is a deformation index derived from the lumbar and femur DXA scans based on a mathematical model called finite element method (FEM) [11]. Finite element models are based on the idea that a complex object can be divided into smaller and simpler elements to simplify problem-solving. In bone structural analysis, finite elements can be used to identify the area most prone to higher stresses, strains and fracture risk.

BSI represents the average equivalent strain in the regions of interest identified by the DXA software. Therefore, it is able to provide a quantitative description of the strain distribution inside the relevant bone segment. BSI being a value index of strain concentration, higher values indicate a higher risk condition, whereas lower values a more resistant bone. In recent clinical studies, BSI appeared to be useful in identifying osteoporotic patients with a higher fracture risk [12] and to characterise patients affected by secondary osteoporosis [13, 14].

Osteoporosis is a multifactorial disease, characterised by a plethora of variables, which are connected in a complicated framework that may be difficult to investigate with classical standard statistical methods. To approach the complexity of the problem, a new mathematical methodology based on artificial neural network (ANN) analysis named Auto-Contractive Map (Auto-CM) has been applied to analyse a database of osteoporotic patients [12, 15]. ANNs are machine learning (ML) algorithms particularly able to compute complex/nonlinear data, as other types of ML algorithms (*e.g*., SVMs, decision tree forests) [16]. ANNs adapt to the problem through progressive approximations, reaching a very high precision and allowing inferences at a single individual level even in the presence of relatively small samples.

In this study, we employed a ML system to address two main questions. The first question regards the possibility of predicting further vertebral fractures by analysing available baseline clinical information. The second question involves the associations among the studied variables, with particular regard to BSI and the absence or presence of further fractures. To do this, we have employed a fourth-generation data mining tool represented by Auto-CM.

## Methods

### Study population

This study is a retrospective longitudinal multicentric study conducted at Fondazione IRCCS Ca’ Granda Ospedale Maggiore Policlinico of Milan, Italy; IRCCS Istituto Ortopedico Galeazzi in Milan, Italy; and IRCCS Policlinico San Donato, San Donato Milanese, Italy.

Female patients were selected among those who attended our densitometric service for routine evaluation of bone density and vertebral fractures. Among them, we enrolled 174 women that fulfilled the inclusion/exclusion criteria. The inclusion criteria were the presence of a dorso-lumbar spine x-ray and both femoral and spine DXA scans performed at the same time of the x-ray. The exclusion criteria were the presence of bone metabolic disorders (except for primary osteoporosis), and any history of traumatic and/or pathological fractures. We also excluded those patients undergoing pharmacological treatments known to interfere with bone metabolism (*e.g*., glucocorticoid therapy), except for osteoporosis treatments. Data had been collected within the time frame from September 2010 to August 2018.

All patients underwent a baseline lumbar spine and femoral DXA scans to quantify femur and lumbar spine bone mineral content (BMC), BMD and BSI, together with a spine x-ray to calculate the Spine Deformity Index (SDI) in order to quantify the severity of vertebral fractures [17]. A fracture was considered as a one-unit increase of SDI. Demographic, anthropometric and clinical data were collected. All patients had imaging follow-up consisting of plain x-rays and a DXA study after a period that lasted from 1 to 9 years (mean 3.34, SD 1.91, median 2.72). For each patient, we evaluated two sets of exams (lumbar and femoral DXA, dorso-lumbar x-ray), one at baseline and one at follow-up.

**Fig. 1:**
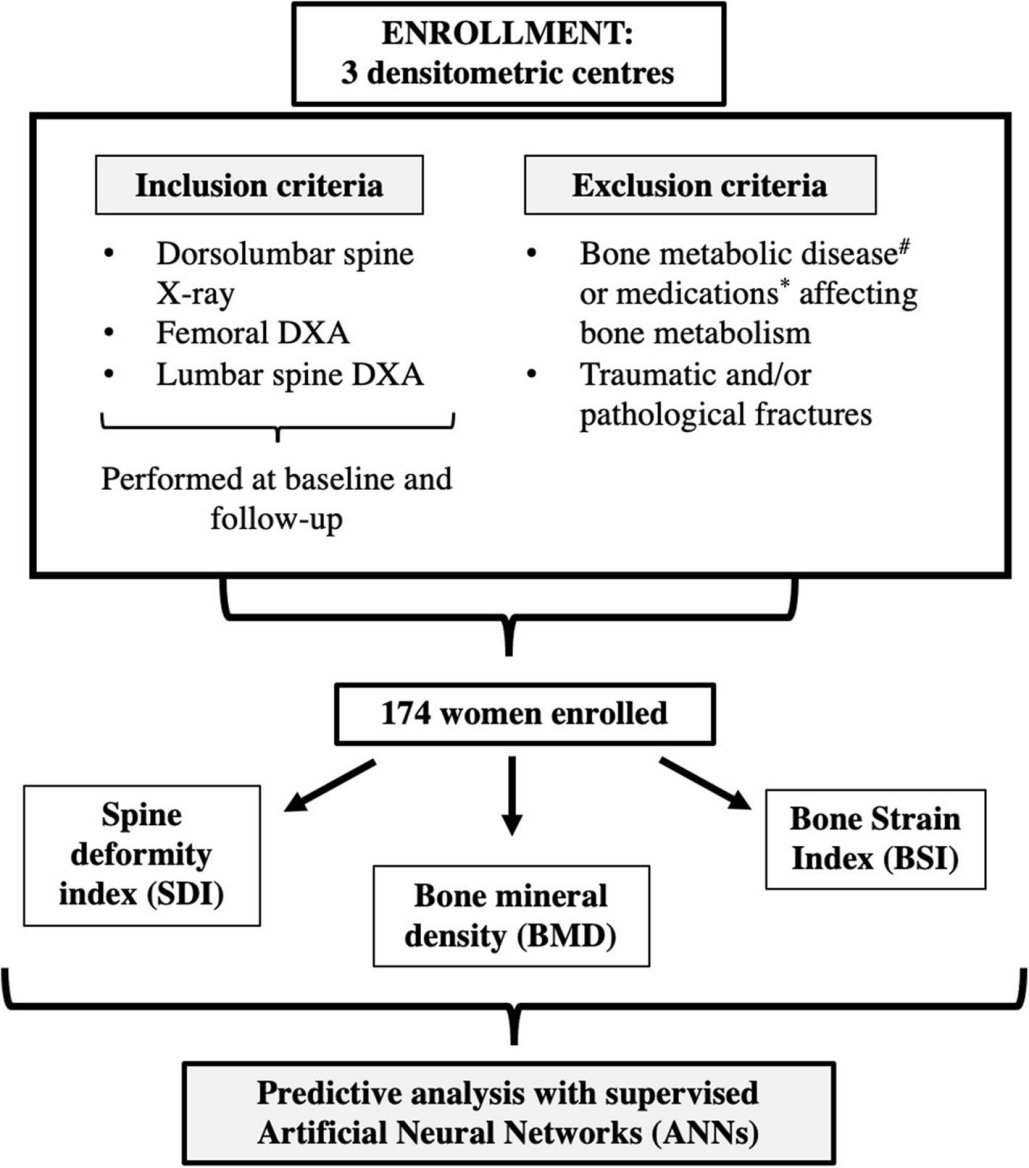
Overview of the study. After applying the inclusion/exclusion criteria, we enrolled a final number of 174 women

Patients gave their written informed consent to the management of their sensitive data for scientific research. Local Ethical Committees’ approval was obtained: Comitato Etico Milano Area 2. Protocol N 2.0 BQ. 265_2017, 13 June 2017 for IRCCS Fondazione Ca’ Granda Ospedale Maggiore Policlinico, Milan, Italy; Comitato Etico San Raffaele; Studio clinico 2.0 BQ, version 4.0, 8 August 2019, for IRCCS Istituto Ortopedico Galeazzi, Milan; and IRCCS Policlinico San Donato, San Donato Milanese (MI). Figure 1 summarises the study flowchart.

### DXA data acquisition

Bone density was assessed by DXA, using a Hologic Discovery A for Fondazione IRCCS Ca’ Granda Ospedale Maggiore Policlinico and IRCCS Policlinico San Donato, and a Hologic QDR-Discovery W for IRCCS Istituto Ortopedico Galeazzi.

Experienced and dedicated technicians performed all the exams according to the International Society for Clinical Densitometry guidelines [18]. All patients underwent an L1–L4 spine scan and hip scan. Those vertebrae affected by fragility fractures were manually excluded from the DXA analysis, in order to avoid fictitious BMD values. Both BMD and BSI were automatically obtained from the same region of interest of the lumbar spine and hip scans.

BSI computation was obtained by an automated software with the use of a constant strain FEA triangular mesh. The pressure applied to the vertebra and hip is specific for each patient and is based on the relationship between forces and the patient’s weight and height, as postulated by Han et al.’s study [19]. The definition of the model’s mechanical properties was done in a stiffness matrix by assigning an elastic modulus depending on the regional BMD values, in accordance with the Morgan's equation [20]. BSI calculation is obtained using a triangular mesh designed on the bone, segmented by the DXA software. In the case of the lumbar spine, the loading force applied to each vertebra is calculated following simulation data provided by Han et al.’s study in standing conditions [19] and uniformly distributed onto the upper facet of each vertebra, whereas the lower side is used as a constraint. In the case of hip scans, loading and constraints follow the indications provided by Terzini et al. [21], with the head and distal femur constrained, and force applied on the greater trochanteric area following a sideway fall condition.

**Fig. 2:**
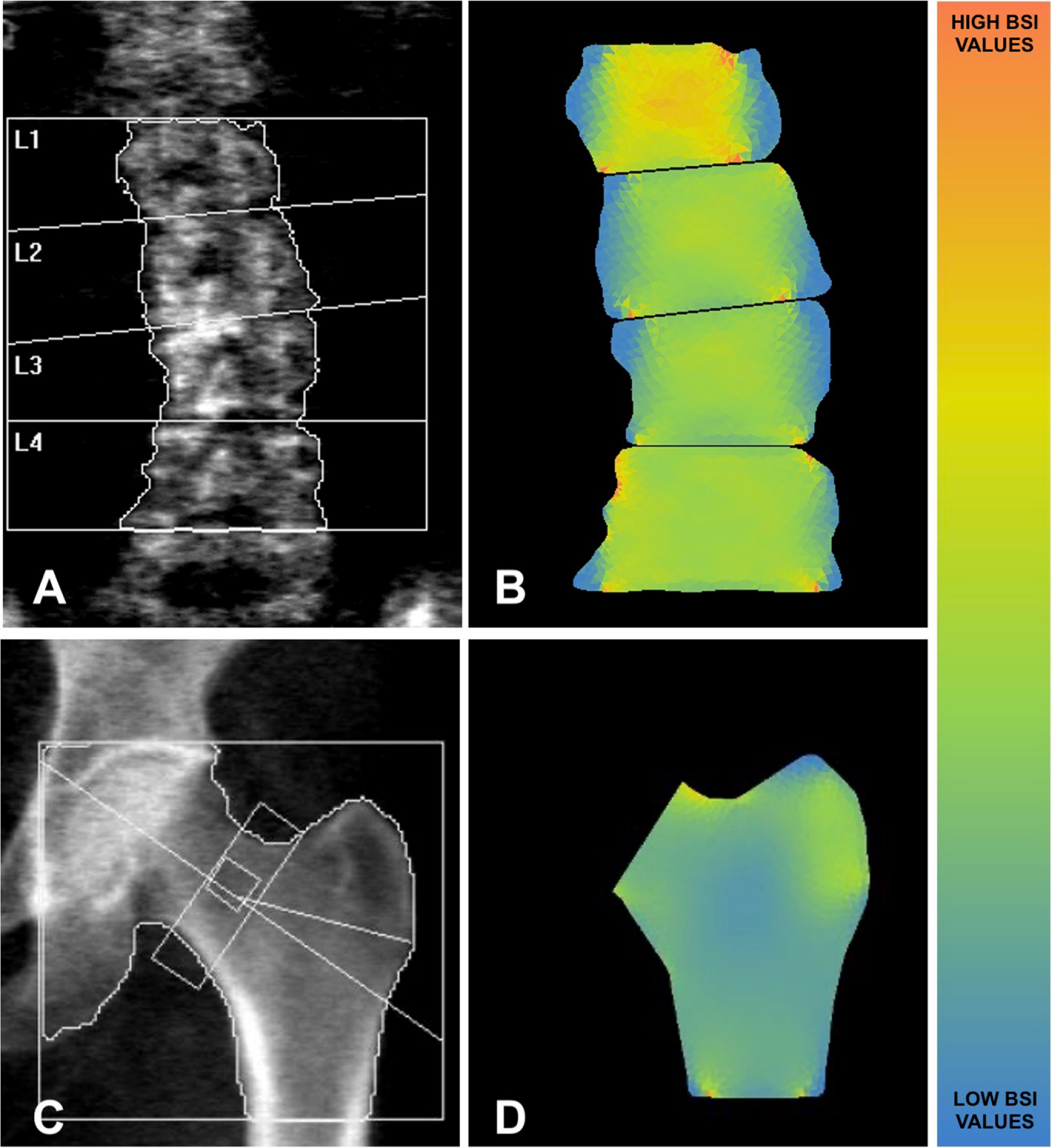
An example of lumbar and femur DXA scans (**A** and **C**, respectively) with corresponding BSI analysis (**B** and **D**, respectively). On the right side, a bar explains the different colours of BSI values, with high BSI reported in red and low BSI reported in blue

Ultimately, the BSI values relate to the average strain inside the specific lumbar vertebra and hip region, obtained with linear elastic analysis and with the assumption that a higher strain level (high BSI) indicates a greater risk condition. Figure 2 shows an example of a DXA scan with the corresponding BSI analysis.

### X-ray data acquisition

Patients underwent an anteroposterior and lateral x-ray of the spine in order to investigate the presence of vertebral fractures (VFs) at the beginning and at the end of the follow-up. The vast majority of spine x-ray examinations were performed in the supine position, and all scans were performed in two projections (frontal and lateral). A radiologist with more than 10 years of experience in osteoporosis imaging assessed all the plain films to evaluate the presence/absence of VFs. We preferred to directly evaluate the images and not the radiological reports as it has been shown that many mild fractures may go unreported [22]. The SDI was calculated using Genant’s semi-quantitative approach by evaluating the vertebrae from T4 to L4; Genant’s visual semiquantitative method consists of giving each vertebra a degree of deformity (mild, moderate and severe) based on the visually apparent degree of vertebral height loss. Fractures are also classified according to the type of deformity (wedge fractures, biconcave fractures, or compressive fractures) [23, 24].

### Predictive analysis with supervised artificial neural networks

Advanced intelligent systems based on the novel coupling of ANNs and evolutionary algorithms have been applied in this study. Supervised ANNs [25] were applied to create a mathematical model to predict the different class outcomes (fracture occurrence versus no fracture occurrence) starting from available clinical and densitometric data. The learning mechanism of the supervised ANNs can make their output coincide with a pre-established target. The presence of learning constraints allows for the supervised ANN output to coincide with the predefined target. The standard formula of these ANNs is *y* = *f* (*x*,w*), where w* represents the set of parameters which best approximates the function.

Data preprocessing was performed using a re-sampling system named TWIST developed by Semeion Research Centre. The TWIST system consists of an ensemble of two previously described systems: T&T and IS [26].

To find out the connectivity traces among variables, a new mapping method was adopted, with the use of a mathematical approach based on an artificial adaptive system; this was done to define the association strength between variables within the dataset (the Auto-CM algorithm). The Auto-CM system is a three-layered architecture fourth-generation unsupervised ANNs able to compute the multi-dimensional association of the strength of each variable with all other variables in a dataset, using a mathematical approach based on recursive non-linear equations. Subsequently to the training phase, the weight matrix of the Auto-CM reflects the warped landscape of the dataset. Therefore, a filter represented by a minimum spanning tree is applied to the Auto-CM system, finally producing a map of the main connections between the variables of the dataset (connectivity map, as detailed in Buscema et al.) [16, 27].

As for previous clinical studies [12, 28–30], after a training phase, the Auto-CM determines the so-called weights of the vectors’ matrix, proportional to the strength of many-to-many connections across all variables, and can be easily visualised by transforming them into physical distances: variables whose connection weights are higher become relatively closer, and vice versa. We transformed the thirteen input variables into 26 input variables, scaled from 0 to 1. Consider, for example, the variable lumbar BMD: absolute natural values range from 0.521 to 1.3. In transformation 1.3, the highest value becomes 1 and 0.521 becomes 0. All other values are scaled to this new range: for example, the value 0.64 becomes 0.15, the value 0.93 becomes 0.53 and so on. In the complement transformation, we permit the system to point out the fuzzy position of the variables, also in accordance with its lower values. With this approach, the projection of the preliminary variables shows high values; on the other side, the complement transformation showed low values of the original variables. We named these two forms as “high” and “low” on the map. This preprocessing is required to compare all the possible variables and to understand the possible links between variables when the values tend to be high or low.

### Statistics

Variables were expressed as mean ± standard deviation (SD) ranges. For comparisons between the groups, an independent-samples *t*-test was used. A two-tailed probability value of 0.05 was considered statistically significant. The linear correlation index between variables was calculated by the Pearson test. A *p*-value < 0.05 was considered to be statistically significant. Statistical analysis was performed with the XLSTAT package 2018.

## Results

Full results are reported in a descriptive table, in a figure with Pearson’s *R* values and in maps that are the usual ANN output analysis. We evaluated *n* = 6 exams for each patient, for a total of *n* = 1,044 exams. The distribution of patients among the centres was as follows: *n* = 53/174 (30.5%) at Fondazione IRCCS Ca’ Granda Ospedale Maggiore Policlinico, *n* = 52/174 (29.9%) at IRCCS Policlinico San Donato and *n* = 69/174 (39.7%) at IRCCS Istituto Ortopedico Galeazzi.

Table 1 shows the characteristic of the studied population. According to the two groups of patients (developing and not developing vertebral fractures) during the follow-up period, the mean values of the following variables resulted in significant differences between women developing VFs at follow-up compared to women without VFs at follow-up: lumbar BMC (*p* = 0.021), lumbar BMD (*p* < 0.005), neck BMC (*p* < 0.005), neck BMD (*p* = 0.006), neck BSI (*p* < 0.005), total femur BMC (TFBMC, *p* = 0.023) and total femur BSI (TFBSI, *p* < 0.005).

**Table 1:** Study population according to the two groups of patients (fractured *versus* non-fractured). *p*-values (last column) refers to the comparison between the group of patients in which a vertebral fracture occurred at follow-up (fracture = yes) and those who did not develop a vertebral fracture (fracture = no)

Characteristic	Whole population (*n* = 174)			Fracture = yes (*n* = 69)		Fracture = no (*n* = 105)		*p*-value
	Mean	SD	Range	Mean	SD	Mean	SD	
Menopause age	48.4	5.1	38–60	48.1	5.4	48.6	4.8	0.5
Weight	60.0	9.6	37–98	58.5	7.9	60.9	10.5	0.4
Age	66.3	9.8	41–88	66.6	10.6	66.1	9.3	0.35
BMI	24.2	3.7	14.82–34.89	24.4	3.7	24.1	3.7	0.8
Lumbar BMC	41.32	9.92	14.96–81.01	37.25	8.10	44.03	10.11	0.021
Lumbar BMD	0.813	0.131	0.525–1.281	0.774	0.090	0.867	0.142	< 0.005
Lumbar BSI	2.298	0.586	1.076–4.299	2.467	0.54	2.031	0.581	0.28
Neck BMC	3.12	0.50	2.11–4.74	2.96	0.37	3.29	0.53	0.001
Neck BMD	0.667	0.091	0.365–0.879	0.603	0.073	0.698	0.102	0.006
Neck BSI	1.929	0.521	0.772–3.991	2.085	0.614	1.821	0.450	< 0.005
TF BMC	24.81	4.46	14.84–38.58	23.71	3.75	25.66	4.73	0.023
TF BMD	0.773	0.11	0.427–1.111	0.752	0.101	0.801	0.115	0.20
TF BSI	1.638	0.402	0.992–3.332	1.751	0.482	1.606	0.325	< 0.005

*SD* Standard deviation, *BMI* Body mass index, *BMC* Bone mineral content, *BMD* Bone mineral density, *BSI* Bone Strain Index, *TF* Total femur

**Fig. 3:**
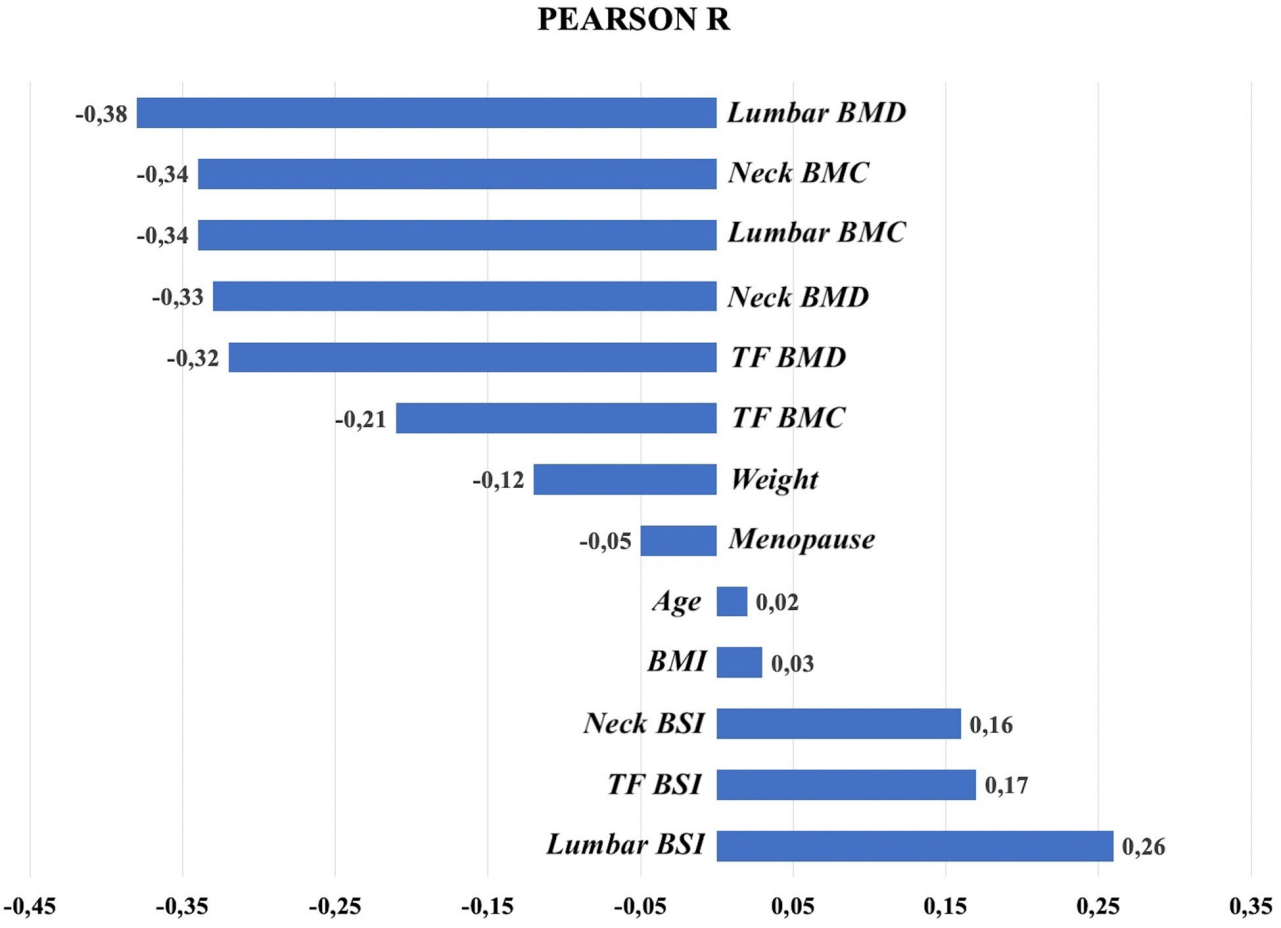
Linear correlation values (Pearson correlation coefficient) between the study variables and the presence of a vertebral fracture at follow-up. BSI, Bone Strain Index; TF, Total femur; BMI, Body mass index; BMC, Bone mineral content; BMD, Bone mineral density

Figure 3 shows the linear correlation values between the study variables and the presence of a VF at follow-up. As expected, BSI parameters predispose to VF at variance with bone mineral content parameters. In any case, the absolute value of Pearson *R* is rather low, and this offers a further rationale for the application of ANNs instead of traditional statistics.

The TWIST system selected the following variables which took part in the modelling by artificial neural networks: weight, age, BMI, lumbar BMC, lumbar BMD, neck BMC, TFBMC, TFBMD and TFBSI. Of note, the system also selected variables with low linear correlation index like weight (0.12) and age (0.02). A global dataset of nine input and two target attributes was thus generated. After that, two optimal subsets were created to apply the training and testing procedure described in the “Methods” section.

**Fig. 4:**
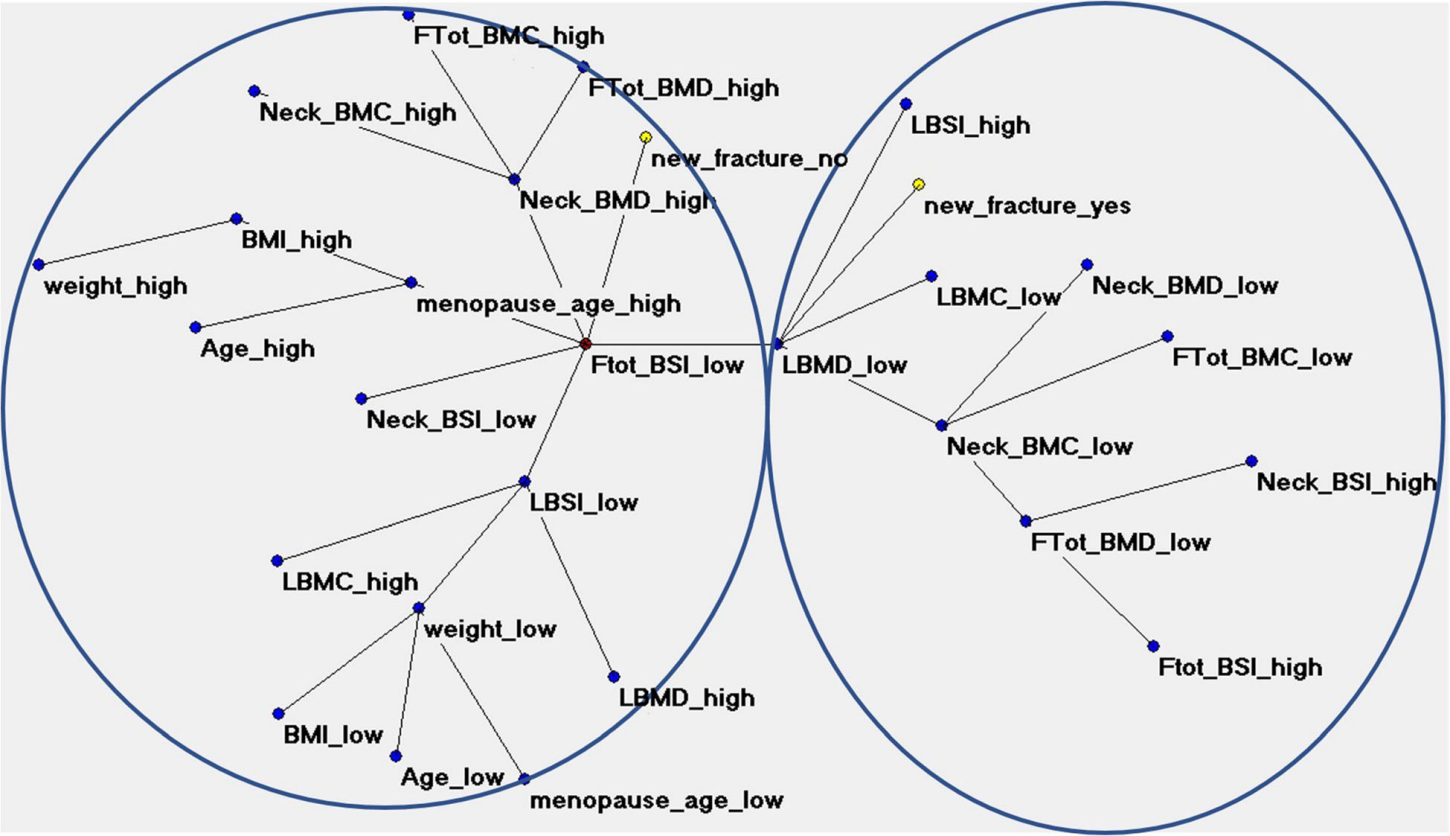
ANN semantic connectivity developed by Auto-CM system, illustrating the connections among variables in the area without a fracture (“new_fracture_no”, on the left side) and the fractured area (“new_fracture_yes”, on the right side). Ftot, Total femur; BMC, Bone mineral content; BMD, Bone mineral density; BSI, Bone strain index; LBMC, Lumbar BMC; LBMD, Lumbar BMD; LBSI, Lumbar BSI. “High” refers to high values, “low” refers to low values

**Fig. 5:**
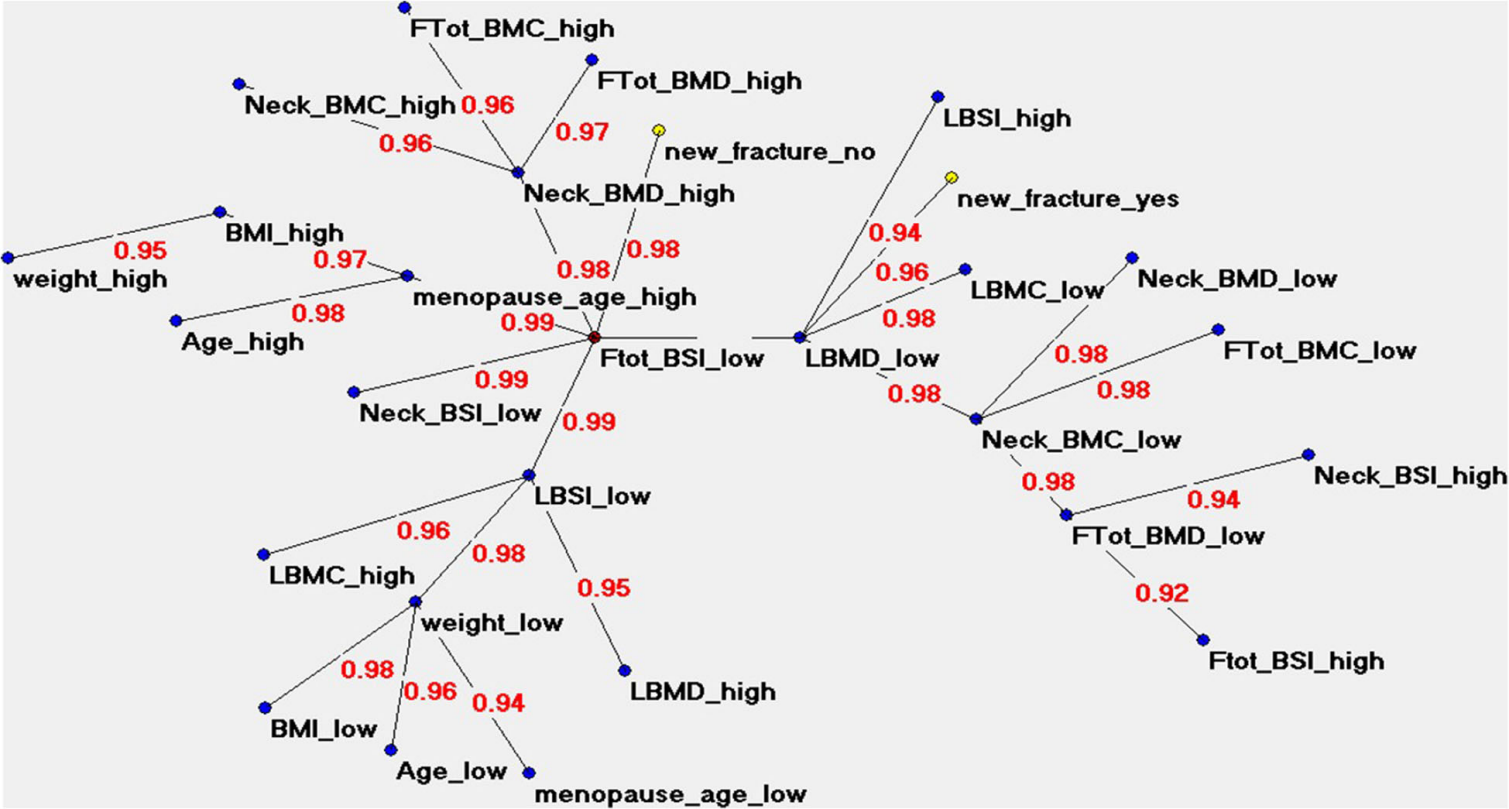
ANN semantic connectivity map showing the different strength values of connectivity as numbers. Ftot, Total femur; BMC, Bone mineral content; BMD, Bone mineral density; BSI, Bone strain index; LBMC, Lumbar BMC; LBMD, Lumbar BMD; LBSI, Lumbar BSI. “High” refers to high values, “low” refers to low values

**Fig. 6:**
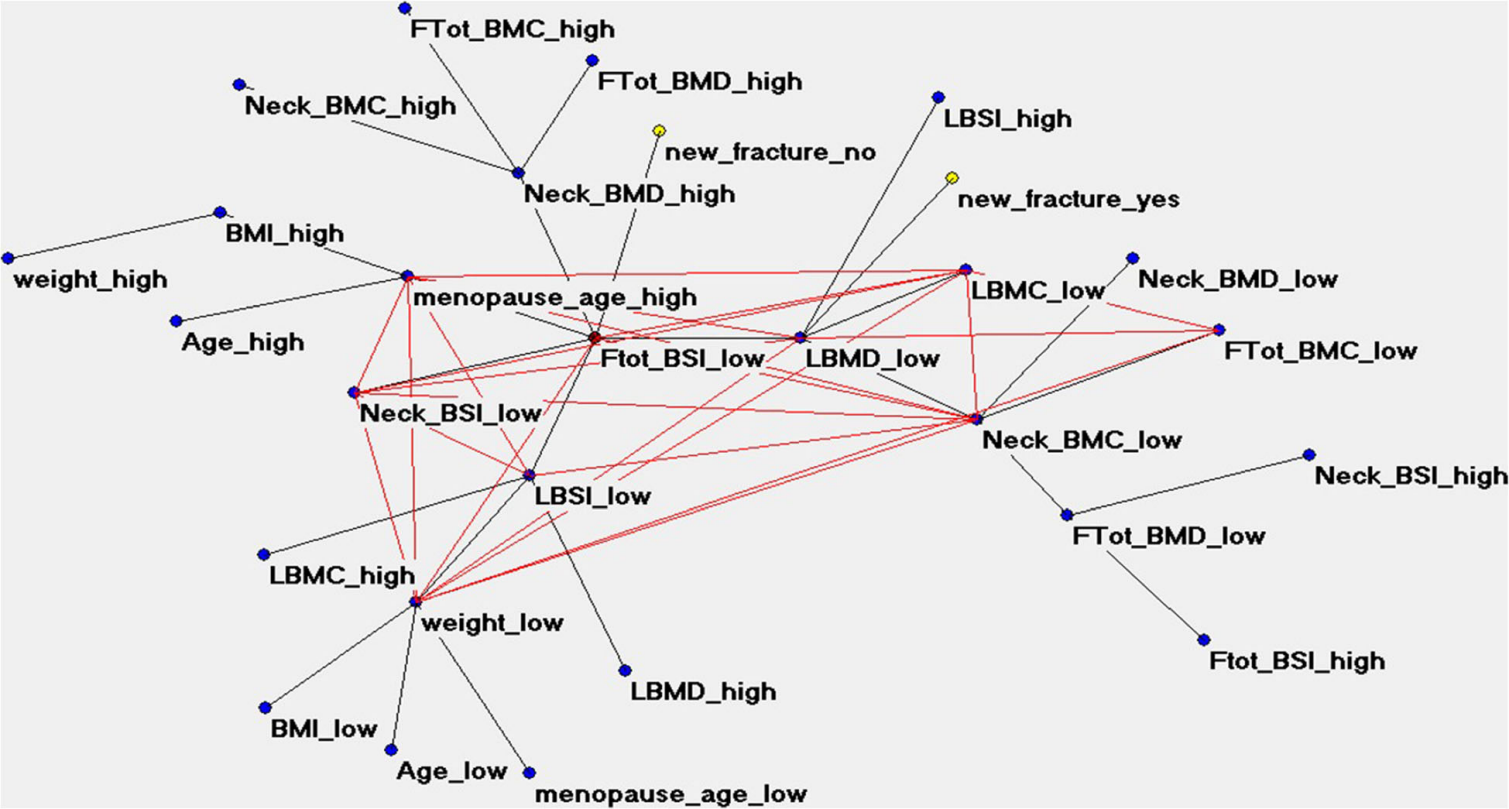
ANN semantic connectivity map with maximally regular graph, a sort of diamond showing the multiple interconnections among variables, suggesting the inherent complexity of the data structure. Ftot, Total femur; BMC, Bone mineral content; BMD, Bone mineral density; BSI, Bone strain index; LBMC, Lumbar BMC; LBMD, Lumbar BMD; LBSI, Lmbar BSI. “High” refers to high values, “low” refers to low values

The performance of artificial neural networks showed an overall high predictive accuracy of 79.56%, as shown in Table 2. This strengthens the added value of the ANN-TWIST pipeline compared to traditional statistical models that reach an average of 60% accuracy. Figure 4 shows the semantic connectivity map developed by the Auto-CM system, illustrating the connections among variables in the area without a fracture (“new_fracture_no”, left) and the fractured area (“new_fracture_yes”, right). Figure 5 shows the same map highlighting the link strength values. Figure 6 shows the same connectivity map with the superimposition of a maximally regular graph depicting a sort of diamond in which there are multiple interconnections among variables, meaning the inherent complexity of the data structure. In these maps “Ftot_BSI_low” (a low value of total femur BSI) is directly connected to the outcome “new_fracture_no” and appears to be a hub of the dense network of connection that links the variables “menopause_age_high”, “LBMD_low” (low lumbar BMD), “Neck_BSI_low” (a low value of neck BSI), “LBSI_low” (a low value of lumbar BSI), “weight_low”, “Neck_BMC_low” and “L_BMC_low (low value of lumbar BMC)”.

**Table 2:** Predictive results using artificial intelligence systems. The results refer to two testing experiments with training-testing A-B and B-A sequences

ANNs	Records	Fracture, yes	Fracture, no	Sensitivity (%)	Specificity (%)	Overall accuracy (%)	AUC
Feed forward back propagation AB	100	36	64	86.11	73.44	79.77	0.781
Feed forward back propagation BA	74	33	41	75.76	82.93	79.34	0.846
**Sum/mean**	**174**	**69**	**105**	**80.93**	**78.18**	**79.56**	**0.824**

*ANN* Artificial neural network

A further analysis has been carried out with a tripartite subdivision of the dataset. The first two subsets with 124 records were used for training-testing experiments while the third subset with 50 records was employed for the validation test. The results obtained are shown in Table 3.

**Table 3:** Predictivity results obtained with tripartite subdivision of the dataset. The first two subsets with 124 records were used for training-testing experiments (*n* = 64 and *n* = 60, respectively), while the third subset with 50 records was employed for validation test

ANN	Records	Fracture yes	Fracture no	Sensitivity (%)	Specificity (%)	Overall accuracy (%)	AUC
Backpropagation sequence AB	64	25	36	82.40	75.60	79.00	0.79
Backpropagation sequence BA	60	25	40	78.50	81.70	80.10	0.82
Backpropagation (validation set)	50	19	29	79.8	78.3	79.05	0.80

## Discussion

Osteoporosis can be assimilated into a complex system with many variables of different clinical significance regarding the prediction of fragility fractures. A significant challenge in the management of osteoporotic patients is to identify, among its many variables, those of the highest weight in determining the fracture event. In fact, the vast number of variables considered in many clinical studies may complicate the comprehension of the clinical meaning of the correlations found [31]. In this context, we used an innovative approach to statistical analysis of our database, which is commonly used in artificial intelligence systems, namely neural network analysis, with a robust predictive tool (TWIST system) and a robust data mining tool, Auto-CM. In our population, we applied supervised neural network modelling on the baseline variables selected by the TWIST system. The analysis highlighted a high performance of ANNs with remarkable results, with an overall predictive accuracy near 80%. We could not find in the literature a performance superior to this in analogue studies focusing on the prediction of VF in patients during a follow-up. Other works used machine learning in order to predict fractures [25], but with some limitations. De Vries et al. presented a low performance of their machine learning approach, reaching an accuracy of 62% [25]. Mehta et al.’s study conducted by machine learning presented a good performance (> 90%), but their study is a cross-sectional one and not a prospective study [32]. Zhang et al. provided an effective approach in the prediction of vertebral strength, suggesting the potential clinical applications for non-invasive vertebral fracture risk assessment [33]. It is interesting to note that, among the selected variables, the TWIST system also included variables with low linear correlation index like weight and age, which would have been almost certainly discarded by linear modelling approaches.

Data mining is an analytic process designed to explore data (usually large amounts of data with complex relationships) in search of consistent patterns and/or systematic relationships between variables, with the aim of discovering elusive trends and associations. The statistical techniques commonly used are principal component analysis (PCA) and agglomerative hierarchical clustering (AHC) [34].

PCA is mathematically defined as an orthogonal linear conversion that transforms the data to a new coordinate system so that the biggest variance of all possible projections of the data arrives to lay on the first coordinate (called the first principal component), the second greatest variance on the second coordinate and so on. AHC is one of the most popular clustering methods which tries to build a hierarchy of clusters with a “bottom-up” approach: each variable is merged with the next most similar variable in a cluster, and the pairs of clusters are then merged as one, moving up the hierarchy until the formation of the so-called dendrogram, which shows the progressive grouping of the variables. It is then possible to gain an idea of a reliable number of classes into which the data can be gathered. These classical statistical techniques have limited power when the relationships between variables are non-linear.

The Auto-Contractive Map Auto-CM system is a fourth-generation unsupervised ANNs able to overcome these limitations, computing the multi-dimensional association of strength of each variable with all other variables in a dataset, using a mathematical approach based on recursive non-linear equations.

Auto-CM has been successfully used in different medical fields indicating ANNs’ utility in the contexts in which many variables interact in complex ways [35, 36]. By applying Auto-CM in this study, we observed a complex relationship between bone quantity and quality DXA variables with high adaptive weight among the connections (Figs. 3 and 4) with the definition of two well-distinct clusters: one characterised by low bone mass (BMC and BMD) and the presence of a fragility fracture, and one characterised by a good bone quality (low BSI) and the absence of a fracture, despite the presence of high menopause age in this group, a well-known risk factor for osteoporotic fracture. Lower values of BSI (which means a good mechanical strength) appear to be a significant positive factor performing better than a high age of menopause in influencing the patient’s fracture prediction.

Regarding the clinical significance of our results, we believe it is important to note that our study points in the direction of a better assessment of individual patients’ fracture risk. Refining the prediction of fracture risk remains the most important challenge in the clinical management of patients with osteoporosis, which unfortunately is a silent disease. In this scenario, BSI may show the potential to represent a new DXA-derived and easily obtainable tool that can improve the identification of those patients at higher risk of fracture.

Our study is not without limitations. Among these, we acknowledge that ANN analysis is particularly suitable for clinical contexts characterised by large samples with numerous variables of different clinical significances. Therefore, the findings of this study have to be validated also in this type of context. The results of this study, obtained with artificial intelligence analysis, could also be validated with a classical statistical approach. Another intrinsic limitation is related to the retrospective design of our study.

Three conclusions arise from this study with artificial intelligence analysis. First, BSI appears to be a useful index of fragility fractured patients identification. In fact, in the semantic map, a low value of BSI (that identify a good status of bone resistance to loads) is very close and directly linked to the absence of fracture. Second, BSI appears able to identify those patients not prone to fragility fractures like other femoral and spine DXA indexes of bone status. Third, ANN Auto-CM is useful to understand the complexity of a chronic multifactorial scenario like osteoporosis and to predict its dramatic consequences, namely the fragility fractures.

## Data Availability

The datasets used and/or analysed during the current study are available from the corresponding author on reasonable request.

## Abbreviations

AHC: Agglomerative hierarchical clustering
ANN: Artificial neural network
Auto-CM: Auto-Contractive Map
BMC: Bone mineral content
BMD: Bone mineral density
BSI: Bone Strain Index
DXA: Dual-energy x-ray absorptiometry
ML: Machine learning
PCA: Principal component analysis
SDI: Spine Deformity Index
TBS: Trabecular Bone Score
TFBMC: Total femur BMC
TFBMD: Total femur BMD
TFBSI: Total femur BSI

## References

[1] NIH Consensus Development Panel on Osteoporosis Prevention, Diagnosis, and Therapy (2001). Osteoporosis prevention, diagnosis, and therapy. JAMA.

[2] Kanis JA, McCloskey EV, Johansson H, Cooper C, Rizzoli R, Reginster JY, on behalf of the Scientific Advisory Board of the European Society for Clinical and Economic Aspects of Osteoporosis and Osteoarthritis (ESCEO) and the Committee of Scientific Advisors of the International Osteoporosis Foundation (IOF) (2013). European guidance for the diagnosis and management of osteoporosis in postmenopausal women. Osteoporos Int.

[3] Marshall D, Johnell O, Wedel H (1996). Meta-analysis of how well measures of bone mineral density predict occurrence of osteoporotic fractures. BMJ.

[4] Melton LJ, Kan SH, Frye MA (1989). Epidemiology of vertebral fractures in women. Am J Epidemiol.

[5] Roux J-P, Wegrzyn J, Arlot ME et al (2010) Contribution of trabecular and cortical components to biomechanical behavior of human vertebrae: an ex vivo study. J Bone Miner Res 25:356–361 10.1359/jbmr.090803 10.1359/jbmr.090803 PMC6956704 19653808

[6] Guglielmi G, Muscarella S, Bazzocchi A (2011) Integrated imaging approach to osteoporosis: state-of-the-art review and update. Radiographics 31:1343–64. 10.1148/rg.315105712 10.1148/rg.315105712 21918048

[7] Manhard MK, Nyman JS, Does MD (2017). Advances in imaging approaches to fracture risk evaluation. Transl Res.

[8] Hans D, Barthe N, Boutroy S, Pothuaud L, Winzenrieth R, Krieg MA (2011). Correlations between trabecular bone score, measured using anteroposterior dual-energy X-ray absorptiometry acquisition, and 3-dimensional parameters of bone microarchitecture: an experimental study on human cadaver vertebrae. J Clin Densitom.

[9] Hans D, Goertzen AL, Krieg M-A, Leslie WD (2011). Bone microarchitecture assessed by TBS predicts osteoporotic fractures independent of bone density: the Manitoba study. J Bone Miner Res.

[10] Pothuaud L, Barthe N, Krieg M-A, Mehsen N, Carceller P, Hans D (2009). Evaluation of the potential use of trabecular bone score to complement bone mineral density in the diagnosis of osteoporosis: a preliminary spine BMD-matched, case-control study. J Clin Densitom.

[11] Zienkiewicz OC, Taylor RLZJ (2005) The finite element method: its basis and fundamentals. Elsevier

[12] Ulivieri FM, Piodi LP, Grossi E et al (2018) The role of carboxy-terminal cross-linking telopeptide of type I collagen, dual X-ray absorptiometry bone strain and Romberg test in a new osteoporotic fracture risk evaluation: a proposal from an observational study. PLoS One 13(1):e0190477. 10.1371/journal.pone.0190477 10.1371/journal.pone.0190477 PMC5755772 29304151

[13] Ulivieri FM, Rebagliati GAA, Piodi LP et al (2018) Usefulness of bone microarchitectural and geometric DXA-derived parameters in haemophilic patients. Haemophilia 24:980–987 10.1111/hae.13611 10.1111/hae.13611 30273987

[14] Rodari G, Scuvera G, Ulivieri FM et al (2018) Progressive bone impairment with age and pubertal development in neurofibromatosis type I. Arch Osteoporos 13(1):93. 10.1007/s11657-018-0507-8 10.1007/s11657-018-0507-8 30151698

[15] Messina C, Piodi LP, Grossi E et al (2020) Artificial neural network analysis of bone quality DXA parameters response to teriparatide in fractured osteoporotic patients. PLoS One 15(3):e0229820. 10.1371/journal.pone.0229820 10.1371/journal.pone.0229820 PMC7065795 32160208

[16] Buscema M, Grossi E (2008). The semantic connectivity map: an adapting self-organising knowledge discovery method in data bases. Experience in gastro-oesophageal reflux disease. Int J Data Min Bioinform.

[17] Sauer P, Leidig G, Minne HW et al (2009) Spine Deformity Index (SDI) versus other objective procedures of vertebral fracture identification in patients with osteoporosis: a comparative study. J Bone Miner Res 6:227–238 10.1002/jbmr.5650060304 10.1002/jbmr.5650060304 2035349

[18] Lewiecki EM, Baim S, Binkley N et al International Society for Clinical Densitometry (2008) Report of the International Society for Clinical Densitometry 2007 Adult Position Development Conference and Official Positions. South Med J 101:735–739 10.1097/SMJ.0b013e31817a8b02 10.1097/SMJ.0b013e31817a8b02 18580720

[19] Han KS, Rohlmann A, Zander T, Taylor WR (2013). Lumbar spinal loads vary with body height and weight. Med Eng Phys.

[20] Morgan EF, Bayraktar HH, Keaveny TM (2003). Trabecular bone modulus-density relationships depend on anatomic site. J Biomech.

[21] Terzini M, Aldieri A, Rinaudo L, Osella G, Audenino AL, Bignardi C (2019). Improving the hip fracture risk prediction through 2D finite element models from DXA images: validation against 3D models. Front Bioeng Biotechnol.

[22] Diacinti D, Vitali C, Gussoni G et al Research Department of FADOI (2017) Misdiagnosis of vertebral fractures on local radiographic readings of the multicentre POINT (Prevalence of Osteoporosis in INTernal medicine) study. Bone. 10.1016/j.bone.2017.05.008 101:230–235 10.1016/j.bone.2017.05.008 28511873

[23] Genant HK, Wu CY, van Kuijk C, Nevitt MC (1993). Vertebral fracture assessment using a semiquantitative technique. J Bone Miner Res.

[24] Crans GG, Genant HK, Krege JH (2005). Prognostic utility of a semiquantitative spinal deformity index. Bone.

[25] de Vries BCS, Hegeman JH, Nijmeijer W, Geerdink J, Seifert C, Groothuis-Oudshoorn CGM (2021). Comparing three machine learning approaches to design a risk assessment tool for future fractures: predicting a subsequent major osteoporotic fracture in fracture patients with osteopenia and osteoporosis. Osteoporos Int..

[26] Buscema M, Grossi E, Intraligi M, Garbagna N, Andriulli A, Breda M (2005). An optimized experimental protocol based on neuro-evolutionary algorithms: application to the classification of dyspeptic patients and to the prediction of the effectiveness of their treatment. Artif Intell Med.

[27] Buscema M, Grossi E, Snowdon D, Antuono P (2008). Auto-contractive maps: an artificial adaptive system for data mining. An application to Alzheimer Disease. Curr Alzheimer Res.

[28] Coppedè F, Grossi E, Lopomo A, Spisni R, Buscema M, Migliore L (2015). Application of artificial neural networks to link genetic and environmental factors to DNA methylation in colorectal cancer. Epigenomics.

[29] Grossi E, Stoccoro A, Tannorella P, Migliore L, Coppedè F (2016). Artificial neural networks link one-carbon metabolism to gene-promoter methylation in Alzheimer’s disease. J Alzheimer’s Dis.

[30] Drago L, Toscano M, De Grandi R (2017). Microbiota network and mathematic microbe mutualism in colostrum and mature milk collected in two different geographic areas: Italy versus Burundi. ISME J.

[31] Eisman JA, Bogoch ER, Dell R et al for the ASBMR Task Force on Secondary Fracture Prevention (2012) Making the first fracture the last fracture: ASBMR task force report on secondary fracture prevention. J Bone Miner Res 27:2039–2046. 10.1002/jbmr.1698 10.1002/jbmr.1698 22836222

[32] Sisman Y, Ercan-Sekerci A, Payveren-Arıkan M, Sahman H (2015). Diagnostic accuracy of cone-beam CT compared with panoramic images in predicting retromolar canal during extraction of impacted mandibular third molars. Med Oral Patol Oral Cir Bucal.

[33] Zhang M, Gong H, Zhang K, Zhang M (2019). Prediction of lumbar vertebral strength of elderly men based on quantitative computed tomography images using machine learning. Osteoporos Int.

[34] Koteluk O, Wartecki A, Mazurek S, Kołodziejczak I, Mackiewicz A (2021). How do machines learn? Artificial intelligence as a new era in medicine. J Pers Med.

[35] Davison KS, Siminoski K, Adachi JD et al (2006) Bone strength: the whole is greater than the sum of its parts. Semin Arthritis Rheum 36:22–31 10.1016/j.semarthrit.2006.04.002 10.1016/j.semarthrit.2006.04.002 16887465

[36] Bouxsein ML, Karasik D (2006). Bone geometry and skeletal fragility. Curr Osteoporos Rep.

